# A real-world disproportionality analysis of sacubitril/valsartan: data mining of the FDA adverse event reporting system

**DOI:** 10.3389/fphar.2024.1392263

**Published:** 2024-08-13

**Authors:** Yiwen Wang, Xuna Liu

**Affiliations:** ^1^ Xi’an International Medical Center Hospital Affiliated to Northwest University, Xi’an, China; ^2^ Shaanxi Provincial People's Hospital, Xi’an, China

**Keywords:** sacubitril/valsartan, adverse event, data mining, FAERS database, pharmacovigilance

## Abstract

**Purpose:**

Sacubitril/valsartan is extensively used in heart failure; however, there are few long-term safety studies of it in a wide range of populations. The aim of this study was to evaluate sacubitril/valsartan-induced adverse events (AEs) through data mining of the U.S. Food and Drug Administration Adverse Event Reporting System (FAERS).

**Methods:**

Reports in the FAERS from the third quarter of 2015 (FDA approval of sacubitril/valsartan) to the fourth quarter of 2023 were collected and analyzed. Disproportionality analyses, including the reporting odds ratio (ROR), the proportional reporting ratio (PRR), the Bayesian confidence propagation neural network (BCPNN), and empirical Bayesian geometric mean (EBGM) algorithms were adopted in data mining to quantify signals of sacubitril/valsartan-associated AEs.

**Results:**

A total of 12,001,275 reports of sacubitril/valsartan as the “primary suspected (PS)” and 99,651 AEs induced by sacubitril/valsartan were identified. More males than females reported AEs (59.95% vs. 33.31%), with the highest number of reports in the 60–70 years age group (8.11%), and most AEs occurred < 7 days (14.13%) and ≥ 60 days (10.69%) after dosing. Sacubitril/valsartan-induced AE occurrence targeted 24 system organ classes (SOCs) and 294 preferred terms (PTs). Of these, 4 SOCs were strongly positive for all four algorithms, including cardiac disorders, vascular disorders, ear and labyrinth disorders, and respiratory, thoracic and mediastinal disorders. Among all PTs, consistent with the specification, hypotension (n = 10,078) had the highest number of reports, and dizziness, cough, peripheral swelling, blood potassium increased, and renal impairment were also reported in high numbers. Notably, this study also discovered a high frequency of side effects such as death, dyspnea, weight change, feeling abnormal, hearing loss, memory impairment, throat clearing, and diabetes mellitus.

**Conclusion:**

This study identified potential new AE signals and gained a more general understanding of the safety of sacubitril/valsartan, promoting its rational adoption in the cardiovascular system.

## 1 Introduction

With the advent of an aging population and changes in lifestyle, heart failure has emerged as a significant public health concern globally (Martin et al., 2024). Sacubitril/valsartan, a novel angiotensin receptor neprilysin inhibitor (ARNI), has garnered attention due to its unique dual mechanism (D’Elia et al., 2017). It not only blocks the action of angiotensin II but also enhances the biological activity of natriuretic peptides through the inhibition of enkephalinase, thereby demonstrating promising efficacy in improving cardiac function and alleviating symptoms (Fernández-Ruiz, 2020).

Since the market approval of sacubitril/valsartan, although several randomized controlled trials (RCTs) have assessed its efficacy and safety in patients with chronic heart failure (Desai et al., 2016; Kim et al., 2022), these trials often have stringent eligibility criteria and are conducted under controlled conditions, which may not fully reflect the actual performance of the drug in a broad patient population. Furthermore, the relatively short duration of clinical trials is insufficient to capture rare or delayed adverse events (AEs) that may occur with long-term drug use.

While there were several prior studies discussing the real-world AEs of sacubitril/valsartan, these studies were of an earlier vintage and were primarily in focused on specific populations, such as the elderly, and specific adverse effects, such as dementia (Gatti et al., 2021; Chen et al., 2023; Lerman et al., 2024). To bridge these gaps, real-world data research on drug safety becomes essential. The FDA’s Adverse Event Reporting System (FAERS) serves as a vital resource, collecting spontaneous reports from healthcare facilities, physicians, and patients, covering a wide range of patient populations and usage scenarios. By analyzing these data in depth, we can gain a more comprehensive understanding of the safety profile and adverse reaction characteristics of sacubitril/valsartan in actual clinical practice. This study aims to conduct a real-world investigation on the safety of sacubitril/valsartan using FAERS data. We will analyze the adverse event reports associated with sacubitril/valsartan, evaluating its safety features in different populations and usage conditions.

## 2 Methods

### 2.1 Data source

Considering the marketing schedule of sacubitril/valsartan, this research downloaded the American Standard Code for Information Interchange (ASCII) report files from the FAERS database from the third quarter of 2015 to the fourth quarter of 2023. The data were processed using R version 4.2.1 (R Foundation for Statistical Computing, Vienna, Austria; http://www.r-project.org).

### 2.2 Data extraction and analysis

**FIGURE 1:**
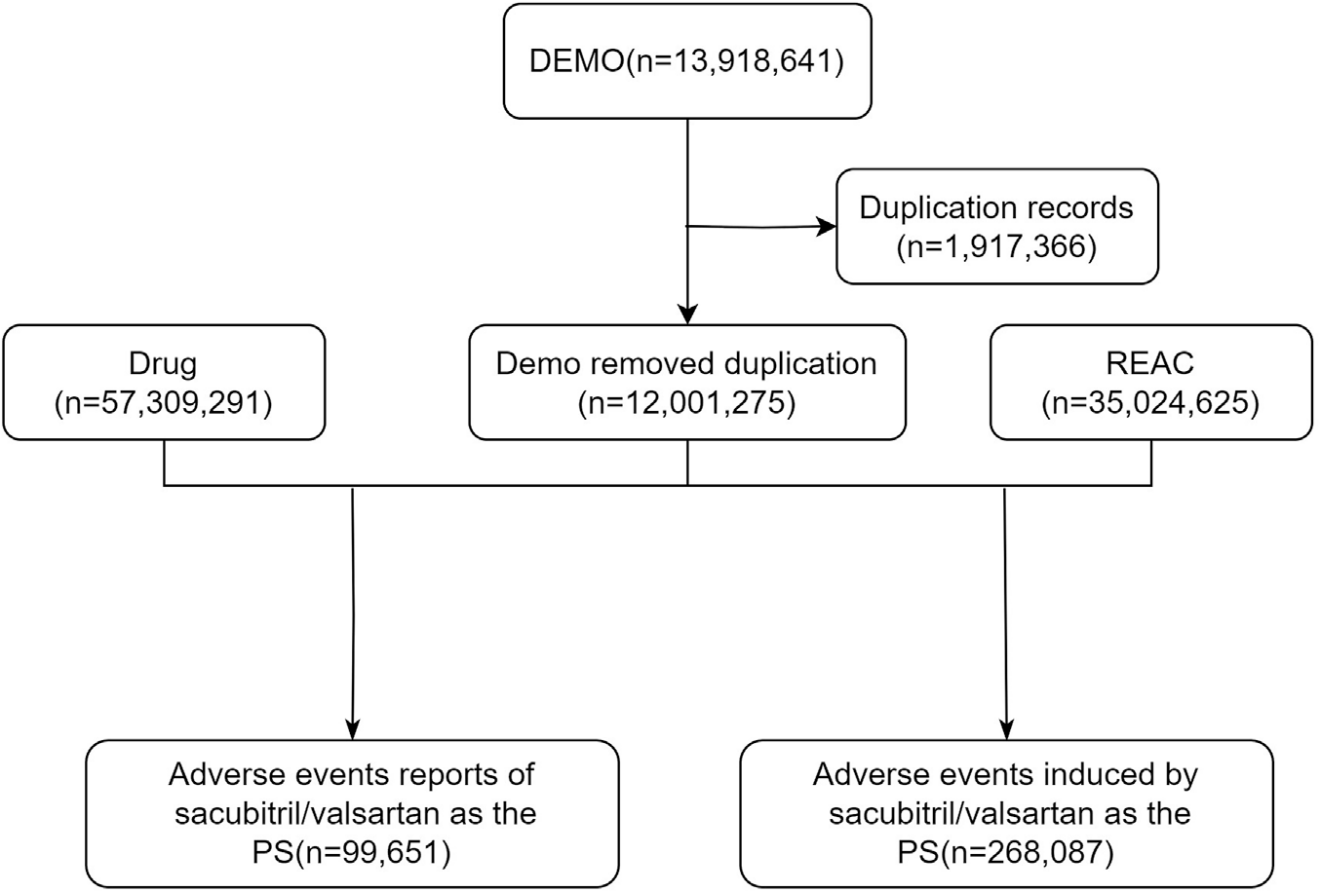
The flow diagram of selecting sacubitril/valsartan-related AEs from FAERS database.

We removed duplicate reports for clean, standardized data. For data with the same case number in the DEMO table, we kept only the most recent report based on date and used the primaryid field to establish relationships between data sets. Drug names were standardized by the Medex_UIMA_1.8.3 system. We extracted reports where the primary drug suspected to be associated with AEs was sacubitril/valsartan, which involved a variety of information such as age, sex, reporter, region, time of reporting, and outcome. The specific flowchart was illustrated in Figure 1 (Foodnotes: DEMO: Demographic; REAC: Reaction; PS: Primary suspect; AE: Adverse event).

This study used a disproportionality measure commonly used in pharmacovigilance studies to capture potential signals between sacubitril/valsartan and AEs. The principle of disproportionality measure is to analyze the degree of association between a drug and an AE by comparing the ratio of frequencies observed in exposed and non-exposed populations using a four-cell scale method (Table 1). In this study, the reported odds ratio (ROR) (Rothman et al., 2004), proportional reporting ratio (PRR) (Evans et al., 2001), Bayesian confidence propagation neural network (BCPNN) (Bate et al., 1998), and empirical Bayesian geometric mean (EBGM) (Dumouchel, 1999) were used to detect drug AE signals. The specific formulas and threshold values are shown in Table 2. The larger the value, the stronger the signal strength and the stronger the association between the target drug and the adverse event. The combined use of multiple algorithms can detect more potentially rare adverse events by adjusting the threshold and variance, and reduce false reports by cross-validation.

**TABLE 1:** Fourfold table of disproportionality method.

	Drug-related AEs	Non-drug-related AEs	Total
Drug	a	b	a + b
Non-drug	c	d	c + d
Total	a + c	b + d	*N* = a + b + c + d

AE, adverse event.

**TABLE 2:** ROR, PRR, BCPNN, and EBGM methods, formulas, and thresholds.

Method	Formula	Threshold
ROR	$\text{ROR}=\frac{a\;/\;c}{b\;/\;d}$	a ≥ 3 ROR ≥ 3 95%CI (lower limit) > 1
	$SE\left(lnROR\right)=\sqrt{\frac{1}{a}+\frac{1}{b}+\frac{1}{c}+\frac{1}{d}}$	
	$95\%CI=e^{\ln\left(ROR\right)\pm 1.96se}$	
PRR	$\text{PRR}=\frac{a\;/\;\left(a+b\right)}{c\;/\;\left(c+d\right)}$	a ≥ 3 PRR ≥ 2 95%CI (lower limit) > 1
	$SE\left(lnPRR\right)=\sqrt{\frac{1}{a}-\frac{1}{a+b}+\frac{1}{c}-\frac{1}{c+d}}$	
	$95\%CI=e^{\ln\left(PRR\right)\pm 1.96se}$	
BCPNN	$\text{IC}=\log_{2}\frac{p\left(x,y\right)}{p\left(x\right)p\left(y\right)}=\log_{2}\frac{a\left(a+b+c+d\right)}{\left(a+b\right)\left(a+c\right)}$	IC025 > 0
	$E\left(\text{IC}\right)=\log_{2}\frac{\left(a+\gamma 11\right)\left(a+b+c+d+\alpha \right)\left(a+b+c+d+\beta \right)}{\left(a+b+c+d+\gamma \right)\left(a+b+\alpha 1\right)\left(a+c+\beta 1\right)}$	
	$V\left(\text{IC}\right)=\frac{1}{{\left(\ln2\right)}^{2}}\left[\frac{\left(a+b+c+d\right)-a+\gamma -\gamma 11}{\left(a+\gamma 11\right)\left(1+a+b+c+d+\gamma \right)}+\frac{\left(a+b+c+d\right)-\left(a+b\right)+a-\alpha 1}{\left(a+b+\alpha 1\right)\left(1+a+b+c+d+\alpha \right)}+\frac{\left(a+b+c+d+\alpha \right)-\left(a+c\right)+\beta -\beta 1}{\left(a+b+\beta 1\right)\left(1+a+b+c+d+\beta \right)}\right]$	
	$\gamma =\gamma 11\frac{\left(a+b+c+d+\alpha \right)\left(a+b+c+d+\beta \right)}{\left(a+b+\alpha 1\right)\left(a+c+\beta 1\right)}$	
	$\text{IC}-2\text{SD}=E\left(\text{IC}\right)-2\;\sqrt{V\left(\text{IC}\right)}$	
EBGM	$\text{EBGM}=\frac{a\left(a+b+c+d\right)}{\left(a+c\right)\left(a+b\right)}$	EBGM05 > 2
	$SE\left(lnEBGM\right)=\sqrt{\frac{1}{a}+\frac{1}{b}+\frac{1}{c}+\frac{1}{d}}$	
	$95\%CI=e^{\ln\left(EBGM\right)\pm 1.96se}$	

### 2.3 Signal filtering and categorization

Preferred terms (PTs) with a reported count of ≥3 were selected for the initial screening in this study (Jiang et al., 2024). Signals were coded, classified, and localized using the MedDRA (Medical Dictionary for Regulatory Activities) PT and the System Organ Class (SOC) to identify the specific SOCs associated with adverse event signals.

## 3 Results

### 3.1 Basic characteristics of sacubitril/valsartan-related AEs

From the third quarter of 2015 through the fourth quarter of 2023, a total of 12,001,275 adverse event reports were obtained from the FAERS database for this study. Of these, 99,651 reports identified sacubitril/valsartan as the primary suspected drug for AEs.

As shown in Table 3, there were more male patients than female patients in the adverse event reports involving sacubitril/valsartan (59.95% vs. 33.31%). Regarding age, although a significant amount of data (79.30%) did not provide information on age, however, the median and quartile age for adverse reactions to sacubitril/valsartan was 68.00 (59.00, 77.00) years in the reports with explicit age data. In further analysis of age, maximum reports (8.11%) were in the age group of 60–70 years followed closely by 70–80 years (7.57%), ≥ 80 years (5.50%), and 50–60 years (5.01%), respectively, thus, the adverse reactions of sacubitril/valsartan were mainly concentrated in the elderly population. In addition, the majority of reports (72.00%) were from consumers rather than healthcare professionals. More than half of the reports were from the United States, accounting for 53.02% of the total. For the route of administration, oral administration accounted for the majority of adverse reactions (51.89%). Concerning clinical outcomes, apart from unspecified serious adverse events, the highest number of adverse events resulted in hospitalization (25.61%), followed by death (24.66%). Finally, the median and quartile of time to adverse events was 14.50 (0.00, 97.00) and the majority of adverse reactions occurred within < 7 days of dosing (14.13%) and ≥ 60 days (10.69%).

**TABLE 3:** Basic information on ADERs related to sacubitril/valsartan from the FAERS database.

Characteristics	Number of events (%)
Sex	
Female	33,198 (33.31)
Male	59,744 (59.95)
Unknown	6,709 (6.73)
Age	68.00 (59.00, 77.00)^a^
< 20	67 (0.07)
20–30	171 (0.17)
30–40	662 (0.66)
40–50	1860 (1.87)
50–60	4,990 (5.01)
60–70	8,077 (8.11)
70–80	7,540 (7.57)
≥ 80	5,482 (5.50)
Unknown	70,802 (71.05)
Reporter	
Consumer	71,751 (72.00)
Physician	15,704 (15.76)
Other health-professional	7,013 (7.04)
Pharmacist	5,099 (5.12)
Unknown	81 (0.08)
Lawyer	3 (0.00)
Reported countries	
United States	52,831 (53.02)
Others	42,491 (42.64)
Japan	2086 (2.09)
Philippines	1,144 (1.15)
Germany	1,099 (1.10)
Route	
Oral	51,707 (51.89)
Others	47,895 (48.06)
Ophthalmic	29 (0.03)
Parenteral	10 (0.01)
Subcutaneous	10 (0.01)
Serious outcomes	
Other serious	25,745 (45.58)
Hospitalization	14,469 (25.61)
Death	13,931 (24.66)
Life threatening	1,556 (2.75)
Disability	751 (1.33)
Required intervention to Prevent Permanent Impairment/Damage	25 (0.04)
Congenital anomaly	12 (0.02)
Adverse event occurrence time - medication date (days)	14.50 (0.00, 97.00)^a^
<7	4,714 (14.13)
7–14	704 (2.11)
14–28	927 (2.78)
28–60	1,204 (3.61)
≥ 60	3,565 (10.69)
Unknown	22,249 (66.69)
Report year	
2015	796 (0.80)
2016	5,859 (5.88)
2017	7,502 (7.53)
2018	11,232 (11.27)
2019	12,763 (12.81)
2020	11,314 (11.35)
2021	15,400 (15.45)
2022	19,002 (19.07)
2023	15,783 (15.84)

^a^ Continuous variables are displayed using the median (first and third quartiles).

### 3.2 Sacubitril/valsartan signal mining

Table 4 shows the AE signal intensity of sacubitril/valsartan at the SOC level. By analyzing the adverse event reports involving sacubitril/valsartan, 24 SOCs were covered by the adverse reactions associated with the drug. Of these, 7 SOCs had case report number > 10,000, ranked in order of general disorders and administration site conditions (n = 48,813), investigations (n = 28,517), injury, poisoning and procedural complications (n = 28,080), respiratory, thoracic and mediastinal disorders (n = 26,065), nervous system disorders (n = 24,958), cardiac disorders (n = 20,739), vascular disorders (n = 15,822), gastrointestinal disorders (n = 10,422). 4 SOCs were strongly positive for all four algorithms, including cardiac disorders (n = 20,739, ROR 3.93, PRR 3.70, IC 1.86, EBGM 3.62), vascular disorders (n = 15,822, ROR 3.11, PRR 2.99, IC 1.56, EBGM 2.94), ear and labyrinth disorders (n = 3,285, ROR 2.76, PRR 2.73, IC 1.43, EBGM 2.70), and respiratory, thoracic and mediastinal disorders (n = 26,065, ROR 2.18, PRR 2.06, IC 1.03, EBGM 2.05). Among them, cardiac organ disorders and vascular disorders had the same SOCs as those corresponding to common adverse reactions in the drug description, indicating a high degree of reliability of the data.

**TABLE 4:** The signal strength of ADEs of sacubitril/valsartan at the SOC level in FAERS database.

System organ class	Case reports	ROR (95% CI)	PRR (95% CI)	χ2	IC (IC025)	EBGM (EBGM05)
General disorders and administration site conditions	48,813	0.98 (0.97, 0.99)	0.99 (0.99, 0.99)	10.64	−0.02 (−0.03)	0.99 (0.98)
Investigations	28,517	1.87 (1.85, 1.89)^a^	1.78 (1.75, 1.82)^a^	10,164.64^a^	0.82 (0.8)^a^	1.77 (1.75)
Injury, poisoning and procedural complications	28,080	0.93 (0.92, 0.94)	0.94 (0.92, 0.96)	135.15	−0.09 (−0.11)	0.94 (0.93)
Respiratory, thoracic and mediastinal disorders	26,065	2.18 (2.15, 2.21)^a^	2.06 (2.02, 2.1)^a^	14,755.93^a^	1.03 (1.01)^a^	2.05 (2.02)^a^
Nervous system disorders	24,958	1.16 (1.14, 1.17)	1.14 (1.12, 1.16)	481.93	0.19 (0.17)	1.14 (1.13)
Cardiac disorders	20,739	3.93 (3.87, 3.98)^a^	3.7 (3.63, 3.77)^a^	40,497.96^a^	1.86 (1.83)^a^	3.62 (3.58)^a^
Vascular disorders	15,822	3.11 (3.06, 3.16)^a^	2.99 (2.93.3.05)^a^	20,840.97^a^	1.56 (1.53)^a^	2.94 (2.9)^a^
Gastrointestinal disorders	10,422	0.43 (0.42, 0.44)	0.45 (0.44, 0.46)	7,521.96	−1.14 (−1.17)	0.45 (0.45)
Infections and infestations	9,686	0.64 (0.62, 0.65)	0.65 (0.64, 0.66)	1937.02	−0.62 (−0.65)	0.65 (0.64)
Musculoskeletal and connective tissue disorders	9,165	0.62 (0.61, 0.63)	0.63 (0.62, 0.64)	2047.04	−0.65 (−0.68)	0.64 (0.62)
Metabolism and nutrition disorders	9,062	1.64 (1.61, 1.68)^a^	1.62 (1.59, 1.65)^a^	2,169.91^a^	0.69 (0.66)^a^	1.61 (1.58)
Psychiatric disorders	8,609	0.55 (0.54, 0.57)^a^	0.57 (0.56, 0.58)^a^	2,975.3^a^	−0.81 (−0.84)	0.57 (0.56)
Renal and urinary disorders	6,619	1.22 (1.19, 1.25)^a^	1.21 (1.19, 1.23)^a^	245.34^a^	0.27 (0.24)^a^	1.21 (1.18)
Skin and subcutaneous tissue disorders	5,692	0.35 (0.34, 0.36)	0.36 (0.35, 0.37)	6,826.56	−1.46 (−1.5)	0.36 (0.35)
Eye disorders	3,415	0.62 (0.6, 0.64)	0.63 (0.61, 0.66)	768.59	−0.67 (−0.72)	0.63 (0.61)
Ear and labyrinth disorders	3,285	2.76 (2.66, 2.85)^a^	2.73 (2.63, 2.84)^a^	3,552.74^a^	1.43 (1.38)^a^	2.7 (2.62)^a^
Neoplasms benign, malignant and unspecified (incl cysts and polyps)	1949	0.22 (0.21, 0.23)	0.22 (0.21, 0.23)	5,388.08	−2.15 (−2.21)	0.23 (0.22)
Immune system disorders	1794	0.53 (0.51, 0.56)	0.54 (0.52, 0.56)	719.37	−0.89 (−0.96)	0.54 (0.52)
Hepatobiliary disorders	819	0.36 (0.34, 0.39)	0.36 (0.34, 0.38)	916.14	−1.45 (−1.55)	0.37 (0.35)
Blood and lymphatic system disorders	786	0.17 (0.16, 0.18)	0.17 (0.16, 0.18)	3,152.04	−2.52 (−2.62)	0.17 (0.16)
Reproductive system and breast disorders	563	0.28 (0.25, 0.3)	0.28 (0.26, 0.3)	1,065.58	−1.84 (−1.96)	0.28 (0.26)
Endocrine disorders	301	0.42 (0.38, 0.47)	0.42 (0.37, 0.47)	234.31	−1.23 (−1.39)	0.43 (0.39)
Congenital, familial and genetic disorders	134	0.17 (0.15, 0.21)	0.17 (0.14, 0.2)	522.16	−2.51 (−2.75)	0.18 (0.15)
Pregnancy, puerperium and perinatal conditions	31	0.03 (0.02, 0.04)	0.03 (0.02, 0.04)	1,036.97	−5.13 (−5.63)	0.03 (0.02)

^a^ Red font indicates that the algorithm was positive.

Of note, ear and labyrinth disorders and respiratory, thoracic and mediastinal disorders were adverse events specific to sacubitril/valsartan and were not mentioned in the drug inserts, which may require further attention and research. Finally, although SOCs were not all positive in the four algorithms, they were never mentioned in the drug labeling and still deserved our notice, including infections and infestations (n = 9,686), musculoskeletal and connective tissue disorders (n = 9,165), psychiatric disorders (n = 8,609), eye disorders (n = 3,415), neoplasms benign, malignant and unspecified (incl cysts and polyps) (n = 1949), immune system disorders (n = 1794).

At the PT level, four algorithms were used in this study to analyze the adverse drug reactions and assess whether they met the various screening criteria, resulting in 294 PTs. The top 30 PTs, sorted according to the number of reports, were shown in Table 5 (sorted by ROR as shown in Supplementary Table S1), and were positive for all four algorithms. The results revealed that hypotension (n = 10,078) had the highest number of reports among all PTs, and consistent with the specification, dizziness, cough, peripheral swelling, blood potassium increased, and renal impairment also had high numbers of reports. Apart from the side effects mentioned in the insert, this study also observed a high frequency of side effects such as death, dyspnea, weight change, feeling abnormal, hearing loss, memory impairment, throat clearing, and diabetes mellitus.

**TABLE 5:** The top 30 signal strength of adverse events of sacubitril/valsartan ranked by the number of case reports at the PTs level in FAERS database.

PTs	Case reports	ROR (95% CI)	PRR (95% CI)	χ2	IC(IC025)	EBGM(EBGM05)
Hypotension	10,078	13.06 (12.79, 13.34)	12.6 (12.36, 12.85)	98,066.64	3.53 (3.5)	11.53 (11.34)
Death	9,061	2.32 (2.27, 2.36)	2.27 (2.23, 2.31)	6,423.1	1.17 (1.14)	2.25 (2.21)
Dyspnoea	7,792	3.22 (3.15, 3.3)	3.16 (3.1, 3.22)	11,303.11	1.63 (1.6)	3.1 (3.04)
Dizziness	7,552	3.6 (3.52, 3.69)	3.53 (3.46, 3.6)	13,412.49	1.79 (1.76)	3.46 (3.39)
Wrong technique in product usage process	7,285	6.07 (5.92, 6.21)	5.93 (5.81, 6.05)	28,619.65	2.51 (2.48)	5.7 (5.59)
Cough	7,266	5.89 (5.75, 6.03)	5.75 (5.64, 5.86)	27,402.5	2.47 (2.44)	5.54 (5.43)
Weight decreased	4,668	3.8 (3.69, 3.92)	3.76 (3.69, 3.83)	9,203.68	1.88 (1.84)	3.67 (3.59)
Cardiac failure	3,421	10.73 (10.36, 11.12)	10.61 (10.2, 11.03)	27,463.62	3.3 (3.25)	9.85 (9.57)
Weight increased	3,196	3.35 (3.23, 3.47)	3.32 (3.19, 3.45)	5,059.83	1.7 (1.65)	3.26 (3.16)
Blood pressure decreased	2,641	10.38 (9.97, 10.8)	10.28 (9.88, 10.69)	20,462.49	3.26 (3.2)	9.57 (9.26)
Myocardial infarction	2,577	5.75 (5.53, 5.98)	5.7 (5.48, 5.93)	9,574.92	2.46 (2.4)	5.5 (5.32)
Inappropriate schedule of product administration	2,565	2.78 (2.67, 2.89)	2.76 (2.65, 2.87)	2,824.29	1.44 (1.39)	2.72 (2.63)
Feeling abnormal	2,382	2.15 (2.06, 2.24)	2.14 (2.06, 2.23)	1,427.11	1.08 (1.03)	2.12 (2.05)
Hypoacusis	2,280	10.14 (9.71, 10.58)	10.06 (9.67, 10.46)	17,220.15	3.23 (3.17)	9.38 (9.05)
Cardiac disorder	2,160	6.13 (5.87, 6.4)	6.09 (5.86, 6.33)	8,766.15	2.55 (2.49)	5.85 (5.64)
Ejection fraction decreased	2,120	40.05 (38.13, 42.06)	39.74 (38.21, 41.33)	60,674.58	4.92 (4.86)	30.35 (29.13)
Memory impairment	2,119	3.28 (3.14, 3.43)	3.26 (3.13, 3.39)	3,251.8	1.68 (1.62)	3.21 (3.09)
Hypertension	2,111	2.39 (2.28, 2.49)	2.37 (2.28, 2.46)	1,654.03	1.23 (1.17)	2.35 (2.27)
Fluid retention	2022	8.76 (8.37, 9.17)	8.7 (8.37, 9.05)	12,891.34	3.04 (2.97)	8.2 (7.89)
Peripheral swelling	1963	2.18 (2.08, 2.28)	2.17 (2.09, 2.26)	1,221.06	1.1 (1.04)	2.15 (2.07)
Cerebrovascular accident	1,689	2.98 (2.84, 3.13)	2.97 (2.86, 3.09)	2,154.55	1.55 (1.48)	2.92 (2.8)
Chest pain	1,664	2.37 (2.25, 2.48)	2.36 (2.27, 2.45)	1,280.98	1.22 (1.15)	2.33 (2.24)
Prescribed underdose	1,653	15.49 (14.72, 16.31)	15.4 (14.52, 16.33)	19,815.24	3.79 (3.71)	13.81 (13.23)
Atrial fibrillation	1,628	3.99 (3.8, 4.19)	3.97 (3.74, 4.21)	3,515.36	1.96 (1.89)	3.88 (3.72)
Throat clearing	1,482	132.09 (122.78, 142.11)	131.36 (121.45, 142.07)	93,211.3	6.01 (5.92)	64.37 (60.55)
Illness	1,249	2.76 (2.61, 2.92)	2.75 (2.59, 2.92)	1,363.3	1.44 (1.36)	2.71 (2.59)
Blood potassium increased	1,210	21.05 (19.8, 22.37)	20.96 (19.76, 22.23)	19,682.35	4.18 (4.09)	18.08 (17.18)
Renal impairment	1,188	3.09 (2.92, 3.28)	3.08 (2.9, 3.27)	1,633.83	1.6 (1.52)	3.03 (2.89)
Cardiac failure congestive	1,168	5.97 (5.63, 6.33)	5.94 (5.6, 6.3)	4,587.93	2.52 (2.43)	5.72 (5.44)
Diabetes mellitus	1,069	3.71 (3.49, 3.94)	3.7 (3.49, 3.92)	2044.09	1.86 (1.77)	3.62 (3.44)

*Four algorithms of all PTs were positive.

## 4 Discussion

This study provided a comprehensive description of post-marketing adverse event reporting in sacubitril/valsartan-treated patients by utilizing data from the FAERS database on AE frequency, reporting rates, and disproportionate reporting. In our study, sacubitril/valsartan showed a higher proportion of adverse events in males, patients with a median age of 68 years, and <7 and ≥60 days after administration. Meanwhile, SOCs that were inconsistent with the specification included ear and labyrinth disorders, and respiratory, thoracic and mediastinal disorders, among others. In addition, among PTs, this study identified a high frequency of side effects such as death, dyspnea, weight change, sensory abnormalities, hearing loss, memory impairment, throat clearing, and diabetes.

The distribution of reported adverse events associated with sacubitril/valsartan indicated that a higher proportion of AEs were reported in male patients, which may be attributed to the predisposition of males to heart failure with reduced ejection fraction (HFrEF) (Lam et al., 2019), the predominant use of sacubitril/valsartan for the treatment of HFrEF, and the different patterns of drug response exhibited by different genders with respect to their lifestyles or genetic factors. Secondly, the analysis of age distribution demonstrated that the highest percentage of AEs was reported in the age group of 60–70 years, and the rate of reporting was also higher in the age group of ≥ 70 years, which may be related to the more frequent use of sacubitril/valsartan in older patients as well as physiologic differences such as decreased renal function and decreased ability to metabolize the drug (Lerman et al., 2024). Therefore, for this specific population, physicians should adequately assess the overall health status of patients before prescribing and closely monitor for possible adverse effects during treatment. In addition, the high number of adverse reactions reported during the initial phase of sacubitril/valsartan treatment suggests that patients may be more sensitive to the drug during the initiation of treatment. Therefore, physicians should intensify follow-up of patients at the beginning of treatment to detect and manage possible adverse reactions in a timely manner to ensure that patients can safely continue treatment.

In the signal mining of sacubitril/valsartan, we observed a number of adverse effects in agreement with the specification. The largest calculated value for the four formulas of adverse effects was cardiac organ disease, followed closely by vascular disorders, which is consistent with the pharmacologic effects of sacubitril/valsartan as a therapeutic agent for heart failure (Ponikowski et al., 2016). Hypotension was the most frequent adverse effect, and several previous studies have demonstrated this outcome (McMurray et al., 2014; Greene et al., 2021). Sacubitril/valsartan contains sacubitril, an enkephalinase inhibitor, and valsartan, an angiotensin receptor antagonist. Sacubitril/valsartan sodium inhibits enkephalinase (neutral peptide endonuclease; NEP) via LBQ657 (the active metabolite of the prodrug sacubitril), while blocking the angiotensin II type 1 receptor (AT1) via valsartan. Increasing the level of peptides degraded by enkephalinase (e.g., natriuretic peptide) by LBO657 and inhibiting angiotensin II I by valsartan produce cardiovascular and renal effects in patients with heart failure (Rivas García and Álvarez-García, 2024). Valsartan inhibits angiotensin III action by selectively blocking AT1 receptors and also inhibits angiotensin II-dependent aldosterone release. Upon initiation of therapy, as a result of the synergistic effect of these mechanisms, blood pressure may decrease significantly in some patients, especially those who are drug-sensitive or who are initiated on higher doses. Therefore, when using sacubitril/valsartan for the treatment of hypertension or heart failure, physicians usually start treatment at a small dose and gradually adjust the dose based on the patient’s blood pressure response, while closely monitoring blood pressure changes to minimize the risk of hypotension. Also, dizziness of the nervous system, cough of the respiratory system, peripheral swelling, elevated blood potassium and renal impairment were reported in high numbers (Senni et al., 2016).

Noteworthy, we discovered some new signals of adverse effects. Respiratory, thoracic and mediastinal disorders, nervous system disorders, gastrointestinal disorders, infections and infestations, musculoskeletal and connective tissue disorders, metabolism and nutrition disorders, psychiatric disorders, skin and subcutaneous tissue disorders, eye disorders, ear and labyrinth disorders, neoplasms benign, malignant and unspecified (incl cysts and polyps), and immune system disorders were not included in the drug insert. Respiratory system in addition to the common cough, dyspnea has a high reporting rate. Dyspnea caused by sacubitril/valsartan may be related to its inhibitory effect on enkephalinase. Enkephalinase is an enzyme capable of degrading a variety of peptides, including endogenous peptides such as bradykinin (Bozkurt et al., 2023). When enkephalinase is inhibited by sacubitril/valsartan, bradykinin levels in the body increase, leading to vasodilation and increased vascular permeability, which may result in fluid leakage into the alveolar space, causing pulmonary edema. Pulmonary edema is a serious medical condition that interferes with normal gas exchange and leads to breathing difficulties (Ware and Matthay, 2005). Thus, when treating with sacubitril/valsartan, physicians will carefully monitor the patient’s respiratory status, especially in patients with a history of lung disease, to prevent potential dyspnea or pulmonary edema from occurring. Adverse effect of hearing loss has been reported in small numbers previously (Gatti et al., 2021) and may be related to adverse events with labeled diuretics used in the treatment of congestive heart failure (Ponikowski et al., 2016). Memory loss in the nervous system may be related to interference with amyloid-β balance in response to sacubitril/valsartan (Singh et al., 2017). The present study detected a controversial finding that sacubitril/valsartan can cause diabetes mellitus adverse effect. Several previous studies have shown that sacubitril/valsartan improves glycemic control in patients with diabetes and HFrEF (Fernández-Ruiz, 2017; Seferovic et al., 2017; Voordes et al., 2022). Although there is insufficient evidence that sacubitril/valsartan directly causes diabetes, angiotensin II is one of the key components of the RAAS, which promotes insulin secretion from pancreatic beta cells (Dominici et al., 2014). By blocking the action of angiotensin II, sacubitril/valsartan may indirectly affect insulin secretion. Overall, the relationship between sacubitril/valsartan and diabetes is not yet clear, but physicians and patients should be aware of any changes that may affect blood glucose levels and take appropriate measures when necessary. In addition, death was the second most commonly reported PT for sacubitril/valsartan, which may be related to the progression of the patient’s disease, as well as confusion with the indications for sacubitril/valsartan (the FAERS database is based on self-reporting), and therefore, the relationship between death and sacubitril/valsartan still deserves further study. These findings expand our understanding of the spectrum of potential side effects of sacubitril/valsartan and remind healthcare professionals to be vigilant for these symptoms in clinical practice, to be attentive to the possibility of systemic reactions in patients, and to perform appropriate investigations and treatments when necessary.

It is crucial to discuss the limitations of this study. The study’s reliance on FAERS, a spontaneous reporting system, inherently introduces reporting bias due to incomplete data capture, potential underreporting, and delayed entries. Second, the lack of a population base of sacubitril/valsartan users prevented the calculation of the incidence of sacubitril/valsartan-related adverse reactions. Third, the FARES database does not assess the severity of adverse reactions, so only qualitative evaluations can be made. Finally, it is difficult to determine causality from such data because adverse event reports do not equate to confirmed causality and may reflect multiple causative factors. The retrospective design and lack of a control group limit the identification of direct causality. The signaling of all adverse events represents only a statistical correlation, and further clinical observations and prospective studies are needed to determine whether a biological causal relationship exists. Nonetheless, the real-world study provides valuable information about the use of sacubitril/valsartan in a broad patient population and remains a very meaningful guide to the clinical use of this drug.

In conclusion, by performing pharmacovigilance analysis of real-world data from the FAERS database, this study provides information on the safety profile associated with sacubitril/valsartan. In addition to cardiac disorders, vascular disorders and renal and urinary disorders covered in the specification, we found ear and labyrinthine disorders, respiratory system, and chest and mediastinal disorders, among others. Additionally, there was a high incidence of side effects such as death, dyspnea, weight change, sensory abnormalities, hearing loss, memory loss, throat clearing and diabetes. Further studies are still needed to establish causality and validate our results.

## Data Availability

The original contributions presented in the study are included in the article/Supplementary Material, further inquiries can be directed to the corresponding author.
