# Supplementary material for: A real-world disproportionality analysis of sacubitril/valsartan: data mining of the FDA adverse event reporting system
Source: Front Pharmacol. 2024 Aug 13;15:1392263. doi: 10.3389/fphar.2024.1392263 (PMC11347302; doi:10.3389/fphar.2024.1392263)
Supplement: Supplementary file 1 [file Table1.docx]

Supplementary Material

# Supplementary Tables

Supplementary Table 1: The top 30 signal strength of adverse events of sacubitril/valsartan ranked by ROR at the PTs level in FAERS database.

| SOC | PTs | Case Reports | ROR(95% CI) | PRR(95% CI) | χ2 | IC(IC025) | EBGM(EBGM05) |
| --- | --- | --- | --- | --- | --- | --- | --- |
| Respiratory, thoracic and mediastinal disorders | Throat clearing | 1482 | 132.09(122.78, 142.11) | 131.36(121.45, 142.07) | 93211.3 | 6.01(5.92) | 64.37(60.55) |
| Investigations | Ejection fraction decreased | 2120 | 40.05(38.13, 42.06) | 39.74(38.21, 41.33) | 60674.58 | 4.92(4.86) | 30.35(29.13) |
| Investigations | Blood potassium increased | 1210 | 21.05(19.8, 22.37) | 20.96(19.76, 22.23) | 19682.35 | 4.18(4.09) | 18.08(17.18) |
| Injury, poisoning and procedural complications | Prescribed underdose | 1653 | 15.49(14.72, 16.31) | 15.4(14.52, 16.33) | 19815.24 | 3.79(3.71) | 13.81(13.23) |
| Vascular disorders | Hypotension | 10078 | 13.06(12.79, 13.34) | 12.6(12.36, 12.85) | 98066.64 | 3.53(3.5) | 11.53(11.34) |
| Cardiac disorders | Cardiac failure | 3421 | 10.73(10.36, 11.12) | 10.61(10.2, 11.03) | 27463.62 | 3.3(3.25) | 9.85(9.57) |
| Investigations | Blood pressure decreased | 2641 | 10.38(9.97, 10.8) | 10.28(9.88, 10.69) | 20462.49 | 3.26(3.2) | 9.57(9.26) |
| Ear and labyrinth disorders | Hypoacusis | 2280 | 10.14(9.71, 10.58) | 10.06(9.67, 10.46) | 17220.15 | 3.23(3.17) | 9.38(9.05) |
| Metabolism and nutrition disorders | Fluid retention | 2022 | 8.76(8.37, 9.17) | 8.7(8.37, 9.05) | 12891.34 | 3.04(2.97) | 8.2(7.89) |
| Cardiac disorders | Cardiac disorder | 2160 | 6.13(5.87, 6.4) | 6.09(5.86, 6.33) | 8766.15 | 2.55(2.49) | 5.85(5.64) |
| Injury, poisoning and procedural complications | Wrong technique in product usage process | 7285 | 6.07(5.92, 6.21) | 5.93(5.81, 6.05) | 28619.65 | 2.51(2.48) | 5.7(5.59) |
| Cardiac disorders | Cardiac failure congestive | 1168 | 5.97(5.63, 6.33) | 5.94(5.6, 6.3) | 4587.93 | 2.52(2.43) | 5.72(5.44) |
| Respiratory, thoracic and mediastinal disorders | Cough | 7266 | 5.89(5.75, 6.03) | 5.75(5.64, 5.86) | 27402.5 | 2.47(2.44) | 5.54(5.43) |
| Cardiac disorders | Myocardial infarction | 2577 | 5.75(5.53, 5.98) | 5.7(5.48, 5.93) | 9574.92 | 2.46(2.4) | 5.5(5.32) |
| Cardiac disorders | Atrial fibrillation | 1628 | 3.99(3.8, 4.19) | 3.97(3.74, 4.21) | 3515.36 | 1.96(1.89) | 3.88(3.72) |
| Investigations | Weight decreased | 4668 | 3.8(3.69, 3.92) | 3.76(3.69, 3.83) | 9203.68 | 1.88(1.84) | 3.67(3.59) |
| Metabolism and nutrition disorders | Diabetes mellitus | 1069 | 3.71(3.49, 3.94) | 3.7(3.49, 3.92) | 2044.09 | 1.86(1.77) | 3.62(3.44) |
| Nervous system disorders | Dizziness | 7552 | 3.6(3.52, 3.69) | 3.53(3.46, 3.6) | 13412.49 | 1.79(1.76) | 3.46(3.39) |
| Investigations | Weight increased | 3196 | 3.35(3.23, 3.47) | 3.32(3.19, 3.45) | 5059.83 | 1.7(1.65) | 3.26(3.16) |
| Nervous system disorders | Memory impairment | 2119 | 3.28(3.14, 3.43) | 3.26(3.13, 3.39) | 3251.8 | 1.68(1.62) | 3.21(3.09) |
| Respiratory, thoracic and mediastinal disorders | Dyspnoea | 7792 | 3.22(3.15, 3.3) | 3.16(3.1, 3.22) | 11303.11 | 1.63(1.6) | 3.1(3.04) |
| Renal and urinary disorders | Renal impairment | 1188 | 3.09(2.92, 3.28) | 3.08(2.9, 3.27) | 1633.83 | 1.6(1.52) | 3.03(2.89) |
| Nervous system disorders | Cerebrovascular accident | 1689 | 2.98(2.84, 3.13) | 2.97(2.86, 3.09) | 2154.55 | 1.55(1.48) | 2.92(2.8) |
| Injury, poisoning and procedural complications | Inappropriate schedule of product administration | 2565 | 2.78(2.67, 2.89) | 2.76(2.65, 2.87) | 2824.29 | 1.44(1.39) | 2.72(2.63) |
| General disorders and administration site conditions | Illness | 1249 | 2.76(2.61, 2.92) | 2.75(2.59, 2.92) | 1363.3 | 1.44(1.36) | 2.71(2.59) |
| Vascular disorders | Hypertension | 2111 | 2.39(2.28, 2.49) | 2.37(2.28, 2.46) | 1654.03 | 1.23(1.17) | 2.35(2.27) |
| General disorders and administration site conditions | Chest pain | 1664 | 2.37(2.25, 2.48) | 2.36(2.27, 2.45) | 1280.98 | 1.22(1.15) | 2.33(2.24) |
| General disorders and administration site conditions | Death | 9061 | 2.32(2.27, 2.36) | 2.27(2.23, 2.31) | 6423.1 | 1.17(1.14) | 2.25(2.21) |
| General disorders and administration site conditions | Peripheral swelling | 1963 | 2.18(2.08, 2.28) | 2.17(2.09, 2.26) | 1221.06 | 1.1(1.04) | 2.15(2.07) |
| General disorders and administration site conditions | Feeling abnormal | 2382 | 2.15(2.06, 2.24) | 2.14(2.06, 2.23) | 1427.11 | 1.08(1.03) | 2.12(2.05) |
